# A Synthesis of the Evidence for Managing Stress at Work: A Review of the Reviews Reporting on Anxiety, Depression, and Absenteeism

**DOI:** 10.1155/2012/515874

**Published:** 2012-02-14

**Authors:** Kamaldeep S. Bhui, Sokratis Dinos, Stephen A. Stansfeld, Peter D. White

**Affiliations:** Centre for Psychiatry, Barts and the London School of Medicine and Dentistry, Queen Mary University of London, London E14NS, UK

## Abstract

*Background. * Psychosocial stressors in the workplace are a cause of anxiety and depressive illnesses, suicide and family disruption. *Methods.* The present review synthesizes the evidence from existing systematic reviews published between 1990 and July 2011. We assessed the effectiveness of individual, organisational and mixed interventions on two outcomes: mental health and absenteeism. *Results.* In total, 23 systematic reviews included 499 primary studies; there were 11 meta-analyses and 12 narrative reviews. Meta-analytic studies found a greater effect size of individual interventions on individual outcomes. Organisational interventions showed mixed evidence of benefit. Organisational programmes for physical activity showed a reduction in absenteeism. The findings from the meta-analytic reviews were consistent with the findings from the narrative reviews. Specifically, cognitive-behavioural programmes produced larger effects at the individual level compared with other interventions. Some interventions appeared to lead to deterioration in mental health and absenteeism outcomes.Gaps in the literature include studies of organisational outcomes like absenteeism, the influence of specific occupations and size of organisations, and studies of the comparative effectiveness of primary, secondary and tertiary prevention. *Conclusions.* Individual interventions (like CBT) improve individuals' mental health. Physical activity as an organisational intervention reduces absenteeism. Research needs to target gaps in the evidence.

## 1. Introduction

Although work provide a range of benefits such as increased income, social contact, and sense of purpose, it can also have negative effects on mental health, particularly in the form of stress. The National Institute of Occupational Safety and Health in the US (NIOSH) [1] estimate the following:

- 40% of American workers reported their job was very or extremely stressful,
- 25% view their jobs as the number one stressor in their lives,
- three fourths of American employees believe that workers have more on-the-job stress than a generation ago.

Given the global recession, financial strain, and job losses, greater work stress might have adverse consequences in UK. The most recent data from the NHS information centre in UK suggest an increase in the suicide rate for the first time since 1998. The number of people committing suicide rose by 329 to 5,706 in 2008. The rate among men increased from 16.8 per 100,000 in 2007 to 17.7 per 100,000 in 2008. This increase is being interpreted by politicians and the public as a consequence of the global and national recession, increased job insecurity, risk of loss of jobs, and also stress at work, where the demands on the existing workforce have increased (The Independent, 18th November, 2010). 

Approximately 11 million people of working age in UK experience mental health problems. 11.4 million working days were lost in UK in 2008/2009 due to work-related stress, depression, or anxiety [2]. There are also indirect costs, for example, through “presenteeism” when employees are at work but are too unwell to function fully [3]. Stress at work also can lead to physical illness, psychological distress and illness, and sickness absence [3, 4]. Stress, depression, or anxiety accounts for 46% of days lost due to illness and are the single largest cause of all absences attributable to work-related illness [5]. Psychosocial work stressors such as job strain, low decision latitude, low social support, high psychological demands, effort-reward imbalance, and high job insecurity have all been implicated as causes of work stress-related anxiety and depressive illnesses [6]. However, psychosocial work stressors can only be tackled by organisational and systemic strategies and policies.

## 2. The Conceptualisation of Occupational Stress

In order to consider the evidence base, there needs to be some agreement on the meaning of work stress. A popular model of stress considers “inputs” such as job characteristics; for example, excess demands, low control, poor social support, adverse life events such as bereavement or divorce, and additional demands outside of work such as carer responsibilities for a dependent relative or spouse [7–10]. Stress has also been recognised by symptoms or “outputs” such as tension, frustration, or emotional distress. An alternative approach is to theorise that stress is a manifestation of the poor fit between a person and their environment [11]. Stress is then seen to arise due to a discrepancy between the inputs and outputs and the mediating appraisal of stress, personal skills to manage it, and environmental demands and rewards. Transactional models, as those proposed by Lazarus [12] and Cox and Ferguson [13], conceptualise stress as something that unfolds over time within a series of transactions between the person and their environment. Stress is, therefore, elicited and maintained by the individual's actions and perceptions as well as the characteristics of their work environment. 

The specific conceptualisations of stress adopted influence the way interventions are constructed to tackle specific mechanisms in order to alter stress and its manifestations. Cahill [14], Cooper et al. [15], and Marine et al. [16] describe categories of stress management interventions that target the individual or the organisation and specify actions at primary, secondary, or tertiary preventive levels (see Table 1) [17]. Individual interventions include stress awareness training or cognitive behavioural therapy for psychological and emotional stress. Organisational interventions are those that affect whole populations or groups of people and include workplace adjustments or conflict management approaches in a specific organisation. Some interventions target both the individual and organisation, for example, policies to secure a better work-life balance and peer-support groups. Primary interventions aim to prevent the causal factors of stress, secondary interventions aim to reduce the severity or duration of symptoms, and tertiary or reactive interventions aim to provide rehabilitation and maximise functioning among those with chronic health conditions [18].

Although preventive interventions are often advocated, what is the evidence of benefit? The evidence of effective interventions to protect individual mental health and reduce organisational absenteeism rates is difficult to summarise in a manner that is of practical relevance. Therefore, the purpose of this paper is to take the highest level of research evidence (systematic reviews providing narrative synthesis or meta-analyses) and synthesise this evidence to identify the key findings and gaps in the literature on the effectiveness of different stress management interventions for preventing anxiety and depression as the main cause of absenteeism. Consequently, this review of systematic reviews focuses on common mental health problems (anxiety, depression) and absenteeism. 

Undertaking a review of systematic review is challenging methodologically for two reasons; there is not a conventional accepted process to produce a meta-review or meta-synthesis across different types of systematic reviews, for different outcomes, and different complex interventions which may defy drawing a singular scientific conclusion that requires all sources of heterogeneity be overlooked [19]. Secondly, the ambition of the review and the form the findings take have, in part, to reflect the subject matter and the types of interventions that are being reviewed. So, for complex interventions for managing stress at work, there will be organizational and individual interventions, and different disciplinary approaches to the task of meta-synthesis of narrative findings. The notion of a meta-synthesis of narrative findings is itself contested by different qualitative research disciplines from which such approaches have evolved [20, 21]. The purpose of this paper is then to draw together literature and findings which are consistent across reviews and methodologically variant studies, where this is possible in order to demonstrate the strength of the findings. However, given the complex nature of interventions to tackle stress at work and that stress itself and mental health are so ill-defined in studies, we also wish to highlight findings that emerge from a critical comparison of reviews; we also wish to highlight the findings that are pertinent to well-defined common mental disorders (anxiety and depressive states); we also wish to acknowledge that narrative synthesis (or meta-synthesis, as it is sometimes called) may reveal complexities in the field of study such that the findings cannot be neatly expressed as a single statement of efficacy or effectiveness, but that interventions might need to be developed to target specific subpopulations. The findings can, thus, signal the methodological issues that future research must tackle. 

## 3. Methods

The review identified all systematic reviews of evidence on stress management interventions in the workplace and summaries, tabulated extracted, and then synthesized the evidence for the relative merits of different interventions. Consistent with previous work, we restricted the review to papers published since 1990, as recency in the literature is important to ensure the evidence is related to contemporary concepts of stress and work, and to ensure the current work conditions are represented in the evidence synthesis, rather than historical work conditions. The databases searched are listed in Table 2.

The search terms used were:

“psychological ill health or anxiety or stress or distress or burnout,”
“stress management or intervention or rehabilitation or prevention,”
“work or job or employee or sick leave or occupation or workplace adjustments or employee assistance programmes.”

### 3.1. Inclusion and Exclusion Criteria

The criteria used for inclusion were

- english language articles,
- reviews published from 1990 to July 2011,
- systematic reviews,
- reviews with data/narrative synthesis,
- meta-analyses.

The articles excluded were

- theoretical and educational reviews,
- those published prior to 1990.

### 3.2. Types of Reviews

The total number of reviews initially retrieved after excluding duplicates was 7845 (see Table 1). Twenty three reviews that met the inclusion criteria included 499 primary studies/publications. Data were extracted using the headings set out in Table 3 by two researchers working independently. A third researcher checked for and resolved any discrepancies with reference to the original publications.

### 3.3. Outcome Domains

The reviewed studies included many outcomes which ranged from physical health measures (e.g., cardiovascular measures) to psychological and psychiatric measures (e.g., well-being, psychological distress, burnout, general mental health, anxiety, depression, stress, psychiatric symptoms, and psychosomatic symptoms) to organisational measures (e.g., employee satisfaction, motivation, absenteeism). In this paper, we focus only on articles reporting, (a) individual outcomes of symptoms of anxiety and depression (including severe stress if measured by a specific rating scale of anxiety and depression) or anxiety and depressive illness formally assessed using specific diagnostic or psychometric measures and (b) absenteeism as an important organisational outcome as this has an economic cost to the employer. 

We included key words of *anxiety* and *depression* and *severe stress* as inclusion criteria, but many studies and reviews are not flagged on this basis, and the findings pertaining to these outcomes are often hidden in tables of results. Piloting showed that searches specifically for anxiety and depression did not easily permit us to identify all studies that might include anxiety and depression as outcomes; this was only possible after reviewing the full-text paper. Thus, we kept our original searches broad in order to be satisfied all such paper that met our inclusion criteria would be included. 

### 3.4. Analysis

Table 3 presents descriptive information on the twenty three reviews including the dates of published studies/papers included in the reviews, the number of published studies/papers, the prevention level (i.e., primary, secondary, and tertiary), whether the interventions were targeting the individual (I) or the organisation (O) level, and which level the outcomes specified: individual mental health (I) and/or absenteeism (O). 

Due to the heterogeneity of the published reviews in terms of the methodology used (i.e., meta-analyses versus narrative synthesis or meta-narratives), the analysis and synthesis of meta-analytic reviews is reported first (see Table 4; 11 reviews), then the narrative synthesis reviews (Table 5; 12 reviews), each annotated to indicate individual and organisational interventions, and individual and organisational outcomes (see Table 3). 

Including narrative reviews permitted evaluation of in-depth information that might be overlooked in meta-analytic reviews, as this information is important for constructing appropriate interventions and implementing them in order to prevent severe stress and anxiety and depression at work. For example, components of an appropriate organisational intervention will be difficult to capture in a meta-analytic review given these interventions will vary between organisations; only in-depth descriptions can capture the components that can then be considered for similar organisational contexts. 

For meta-analyses, the effect sizes and original conclusions are presented, along with the outcomes used, where these were reported (Table 4). For narrative reviews, we present the key narrative conclusions (or evidence summary statement), along with the number of studies finding improvement (↑), deterioration (↓), or no effect (*↔*). This was done for the same two outcomes: mental health and for absenteeism (Table 5).

Judgements about the number of studies finding a positive, negative or no effect in the narrative synthesis were challenging, as many studies tended to use words such as stress, psychological distress, psychosomatic disorders interchangeably, and negative findings may not have been reported. We only rated studies as having effects on mental health (anxiety and depression), where it was clear they had used a specific measure of mental disorders or severe stress either alone or as part of a composite measure of mental health and well-being. Where there was doubt, we did not include the study in the data. This is an advance on existing reviews which tend to group all types of stress, including that associated with anxiety and depression, and other types of measures of stress such that the findings are interpreted with reference to a large number of emotional and health states. We felt this approach would not permit us to isolate the findings of relevance to the preventing common mental disorders which are the most important cause of sickness-related absenteeism.

## 4. Results

Eleven reviews included meta-analyses [16, 22–31]; 12 included a systematic or literature review [32–43] with meta-narrative conclusions (see Table 5). 

As set out in Table 3, of the twenty three reviews, four reported on *individual interventions only* (three with a meta-analysis) [26, 27, 31, 36]; three of these assessed their impact on individual and organisational outcomes [26, 31, 36], whilst the other one assessed impact on individual outcomes only [27]. There were three reviews that examined the effectiveness of only *organisational interventions *[24, 32, 40]. Of these, Parkes and Sparkes [40] and Bond et al. [24] reviewed organisational outcomes, whereas Egan et al. [32] reported on individual outcomes.

Six reviews included studies that looked *separately at individual and organisational interventions* in the same studies [16, 37, 39–42]. Of these, Mimura and Griffiths [39] reported only on individual outcomes, the rest reported on both individual and organisational outcomes. The remaining seven reviews assessed *interventions at both individual and organisational levels *[23, 25, 29, 30, 33–35]. Of these, one looked only at organisational outcomes [34], and one looked at individual outcomes [28]. There were no studies that assessed interactions between the two levels of outcome. 

### 4.1. Reviews Reporting Meta-Analysis of Effect Sizes

Eleven reviews [16, 22–31] reported effect sizes from meta-analyses (Table 4) on mental health and absenteeism. The overall impression from the meta-analytic reviews is that the effect size is greater at the individual level for individual interventions compared with organisational interventions, and that organisational or mixed interventions can also impact on the mental health of individuals.

### 4.2. Individual Outcomes: Mental Health

Of these eleven reviews, six showed that *individual interventions* lead to benefit on individual mental health outcomes [16, 23, 25–27, 31]. Five reviews of *organisational interventions* [16, 23, 25, 28, 30] together showed mixed evidence of benefit on individual outcomes; thus Richardson and Rothstein [23] and van der Klink et al. [25] showed no benefit, whilst Marine et al. [16], Martin et al. [28] and van Wyk and Pillay-Van Wyk [30] showed some benefit. Richardson and Rothstein [23] and van der Klink et al. [25] also reviewed *mixed interventions*, both of which showed benefit at the individual level on mental health status. 

### 4.3. Organisational Outcomes: Absenteeism

Four reviews found *individual interventions* did not impact on absenteeism [23, 25, 28, 30]. There was mixed evidence of benefit from *organisational interventions *on absenteeism. Parks and Steelman [22] and Bond et al. [24] found some evidence of benefit, whereas Richardson and Rothstein [23] and van der Klink et al. [25] found no benefit. However, Conn et al. [29] showed clear benefit of organisational physical activity interventions on absenteeism. There were no studies of *mixed individual-organisational interventions* and impact on absenteeism. 

### 4.4. Reviews Reporting Narrative Conclusions

The overall conclusions from the narrative reviews support the findings from the meta-analyses that individual interventions do provide benefit at an individual level and reduce symptoms of anxiety and depression and stress, but individual interventions do not impact on absenteeism. However, organisational interventions impact at both individual and organisational levels. There are numerous studies of benefit on mental health outcomes, whereas benefit on absenteeism is mainly reported in one review [33] including a number of high quality studies (Table 5). Worryingly, some interventions appeared to lead to deterioration in mental health [16, 32–35] and absenteeism [33, 36] outcomes (see Table 5). For example, Marine et al. [16] identifies smoking cessation to be associated with depression. Although not directly mapping on to absenteeism, preliminary evidence from Cancelliere et al. [43] suggested that some workplace health promotion programmes can reduce presenteeism (being at work whilst unwell). Presenteeism correlated with being overweight, a poor diet, a lack of exercise, high stress levels, poor relationships with coworkers and management. 

### 4.5. The Effectiveness of Specific Interventions

The different types and components of interventions, and whether they are primary, secondary, or tertiary preventive interventions, are set out in Table 3. The majority of studies were of primary prevention. The meta-analytic reviews found that cognitive behavioural programmes consistently produced larger effects at the individual level compared to other types of interventions (e.g., relaxation). Cognitive behavioural programmes were also suggested to be more effective by some of the narrative reviews [27, 31, 34–36] as well as by some of the meta-analyses [23, 25]. 

Murphy [36] found that multimodal interventions (or combination strategies), which involved CBT produced the most consistent, significant results; a result which was not supported by one meta-analytic review [25]. Overall, the reviews suggested that organisational level interventions are too scarce and there is also a lack of studies that assess organisational-level outcomes. However, two meta-analytic reviews [22, 29] found that participation in organisational wellness programmes was associated with decreased absenteeism and increased job satisfaction. These were the only meta-analytic reviews of organisational based interventions and organisational-level outcomes. Finally, there are insufficient studies to comment on the potential complementarity of interventions that operate at primary, secondary, and tertiary prevention levels [33]. Four studies investigated both primary and secondary prevention but not their interaction [23, 27, 33, 34].

## 5. Discussion

As anticipated, the evidence was in complex form. Our methods of isolating findings related to anxiety and depression, and partitioning the tabulation and extraction and synthesis by individual/organisational interventions and outcomes provides a rich, complex but authentic picture of the evidence base. There are indications for which interventions are effective and also gaps in the evidence. Reviews had to take account of many interventions that differed by their components, mode of delivery and whether they targeted individuals or organisations. This made it difficult for all of the reviews to compare benefits from any single intervention across a number of studies, except for CBT or physical activity. There were also many different outcome measures for assessing anxiety and depression, and many proxy measures of mental health, sometimes without clarity about which outcomes were used in the meta-analyses. In part, these were not specified due to the way multiple outcomes were handled in the analysis. The reviews used standardised differences including mean differences and mean effect sizes, and standardised differences and means. Using a consistent set of outcomes to measure anxiety and depression in future primary studies will ensure that future reviews and meta-analyses can overcome these challenges, such that different intervention, of varying complexity and modes of delivery, might be compared more directly for impacts on absenteeism and on anxiety and depression and interactions between the individual and organisational impacts. 

Overall, individual interventions show larger effects compared with organisational interventions or mixed interventions; benefits are seen mainly at the individual level although some studies do show organisational benefits. Given that anxiety and depression are common, and mostly account for sickness absence, it is important to develop an evidence base that is specific to these manifestations of mental distress and illness, with an agreed range of acceptable outcome measures and for interventions that prevent and treat anxiety and depression promptly, as well as encourage early return to work. A small improvement in sickness absence statistics might yield substantial benefits for business viability and provision of services. Standardised methods to measure presenteeism [43] are needed. The only organisational intervention to show convincing effects on absenteeism was physical activity programmes [29], but mental imaging, CBT, and in vivo exposure, each have a useful role, especially in secondary prevention. Although better quality studies should be given greater weight, the quality of individual primary studies was selectively reported, making it difficult to know whether the positive findings reflected better quality studies; certainly, CBT and physical activity interventions are more well defined than say stress management standards or management practices or stress inoculation. Even counselling can take many forms, and there is not a standardised process. Similarly, the duration of the interventions and timing of measurement of outcomes was not a characteristic on which reviews drew conclusions; we were unable to draw any metaevidence about timing unless we had looked at primary studies. Strikingly, although many reviews on face value were reviewing the same evidence, the reviews did not all identify the same primary studies, and therefore did not always reach the same conclusions; our meta-review, for the first time, brings together all of the strongest findings. We reviewed 23 reviews, after identifying 7845 potential publications for inclusion. These included 499 primary studies; the majority of reviews made the point that drawing metanarrative or meta-analytic conclusions was difficult because of this diversity in outcomes, intervention, and methods. Had we undertaken a review of 499 primary studies, it is likely we would draw the same conclusions. 

Management skills training, and support for staff, along with methods to cope with work stress all seem relevant components, but the review was not convincing about a positive benefit of these and where positive impacts were seen at individual levels [16, 28]; the effect could not entirely be attributed to improved management standards or working relationships. There has been insufficient research on organisational interventions. These studies are difficult to design and implement and require further research. On the other hand, more and more interest has been generated towards health promotion in the workplace (e.g., exercise) and encouraging individuals to take ownership of health risk behaviours and decisions about health, well-being, and family outside of work. This may be promising, as it requires the workforce to maintain healthy lifestyles generally and within that context to consider work stress rather than consider work as the only venue for health interventions. Organisational measures to increase physical activity show promising results [43]. 

This review suggests that there is lack of evidence in comparing the relative effectiveness of stress management interventions that operate at both individual and organisational levels, or interventions that encourage an interactive or systemic effect, yet this might yield greater benefits at both levels. 

However, there are still a number of evidence gaps. More research is needed in the private sector and in smaller companies as well as research comparing different job types such as education and healthcare to examine whether they respond to the same or different intervention techniques. Similarly, research needs to take into account factors such as socioeconomic status, duration of any effects of interventions, and cost effectiveness. Selection bias may be an important explanation for our findings. For example, organisations with the most stressful work environments are less likely to participate in research as opposed to organisations with little stress amongst employees. Consequently, organisations with low baseline stress levels would make any effects from targeted interventions more difficult to capture. However, preliminary support was found in one meta-analytic review that interventions conducted with employees at high levels of baseline stress appeared to be at least as effective as interventions conducted with employees at low levels of baseline stress [25]. What works for whom and the maintenance of these effects need further research [32]. 

Finally, there is a relative lack of studies with clinically referred employees. We did find more of these in more recent years (since 2008) and also reviews of health care workers and law enforcement officers who perhaps need specific attention given the unique circumstances and stressors to which they are exposed at work. The few methodologically rigorous studies that have been conducted with patients have not included *nontreatment* control groups but have compared 2 treatment types. More work might, therefore, be undertaken on populations at risk using secondary and tertiary prevention interventions. 

## 6. Conclusions

CBT was the most effective individual targeted intervention for individual outcomes. Encouragement of physical activity at an organisational level seems to reduce absenteeism. Interventions need to be developed that can provide consistent and stronger effects on organisational outcomes such as absenteeism. There were a number of gaps in the literature, particularly studies investigating the influence of specific occupations, and different sized organisations, different sectors of organisations (public, private, and not for profit). Studies of management practices seemed not to show strong effects, but there are still insufficient studies in this area. There were few studies of secondary and tertiary prevention. 

## Figures and Tables

**Table 1:** Model for categorising stress management interventions (adapted from de Jonge and Dollard) [17].

Level	Primary prevention	Secondary prevention	Tertiary prevention	Outcome measures
Organisational	Improving work content, fitness programmes, and career development	Improving communication and decision making and conflict management	Vocational Rehabilitation and outplacement	Productivity, turn-over, absenteeism, and financial claims

Individual and Organizational interface	Time management, improving interpersonal skills, and Work/home Balance	Peer support groups, coaching, and career planning	Posttraumatic stress assistance programmes and group psychotherapy	Job stressors such as demands, control, support, role ambiguity, relationships, change, with burnout

Individual	Pre-employment medical examination and didactic stress management	Cognitive behavioural techniques and relaxation	Rehabilitation after sick leave, disability management, case management, and individual psychotherapy	Mood states, psychosomatic complaints, subjective experienced stress, physiological parameters, sleep disturbances, and health behaviours

**Table 2:** Databases searched.

Medline 1950 to November Week 3 2008	(*N* = 2, 470)
PsychInfo 1806 to January Week 2 2009	(*N* = 1, 911)
Embase 1980 to 2009 Week 02	(*N* = 2, 313)
Cochrane database of systematic reviews 4th quarter 2008	(*N* = 110)
ACP Journal Club 1991 to December 2008	(*N* = 12)
Cochrane Central Register of Controlled Trials 4th quarter 2008	(*N* = 432)
Cochrane Methodology Register 4th quarter 2008	(*N* = 3)
Allied and Complementary Medicine 1985 to January 2009	(*N* = 335)
British Nursing Index 1985 to January 2009	(*N* = 41)
Health management information consortium October 2008	(*N* = 218)

**Table 3:** Summary of review papers.

Author (search dates)	Research aim or question	Prevention level	Intervention	Outcome	Type of review	Interventions reviewed	No. of Studies
Parks and Steelman 2008 (1980–2005)	Effectiveness of organisational wellness programmes for absenteeism and satisfaction (Publications 1980–2005)	PP	O	O	Meta-analysis	Fitness organisation wellness programmes Comprehensive organisational wellness programmes	15 papers + 2 dissertations 10 on absenteeism

Richardson and Rothstein 2008 (1976 onwards)	Effectiveness of stress management interventions: What works for whom? What has been learned and what is needed next? Includes effectiveness of SMI target by outcomes. Publications in English from 1976 onwards. updates van der Klink, 2001	PP SP PP+SP	I, O I+O	I, O (6 studies), I+O	Systematic with meta analysis	Interventions were categorised into: CBT, relaxation, organisation (support groups), multimodal, and alternative (biofeedback, and personal skills training)	38 papers 4 on absenteeism, 11 on mental health, 13 on anxiety, and 14 on stress

Egan et al. 2007 (up to November 2006)	Whether organisational-level interventions designed to increase employee participation/control lead to health effects predicted by the DCS model	PP	O	I	Systematic	Problem solving committees, employee representatives, delegation of more control of work scheduling, work hours and training, smaller teams with subsupervisors	18 8 on mental health, 4 on stress, 5 on absenteeism, 1 on psychological distress, 1 on psychiatric symptoms, 4 on depression, and 3 on anxiety

Lamontagne et al., 2007 (1990–2005)	Effectiveness of interventions categorised as primary, secondary and tertiary Not a meta-analysis of effect sized, but a narrative review	PP SP TP	O, I, O+I	O, I, O+I	Systematic review of job stress interventions between 1990–2005	Interventions for physical work environment (e.g., noise level), organisation (e.g., work redesign, workload reduction) individual (e.g., coping skills training), and organisation-individual interface (e.g., support group)	90 Not reported

Bond et al. 2006 (1989–2004)	Does increasing employee control and workplace reorganisation (HSE Management standards) affect business outcomes such as performance, absenteeism, and turnover?	—	O	O	Systematic review of interventions	Interventions to increase control including job redesign, steering or focus groups to identify ways that employee control could be increased	5 4 on absenteeism

Marine et al. 2006 (up to May 2005)	Evaluate effectiveness of work and person-directed interventions in preventing stress at work in healthcare workers	PP	O, I	I, O	Cochrane collaboration systematic with meta-analysis and qualitative synthesis	Person directed interventions included cognitive behavioural therapy, relaxation, music making, touch therapy/massage, multicomponent interventions consisting of mindfulness-based stress reduction, education, and exercises to enhance communication skills, stress reactivity and self-compassion and practical skills. Work directed interventions included role playing, experiential exchanges intended to improve attitudes, communication skills, mobilising support from colleagues, learning participatory problems solving and decision making, innovations in nursing delivery via changes in work organisation, knowledge and skills training, and support from supervisors	19 7 on stress, 4 on anxiety, and 1 on absenteeism

The British Occupational Health Research Foundation, 2005 (up to April 2004)	(1) What is the evidence for preventative programmes at work and what are the conditions under which they are most effective? (2) For those at risk what is most effective to enable them to remain at work? (3) What is effective to support rehabilitation and return to work?	PP SP TP	O+I	O	Systematic review	Coworker support group, stress inoculation training, counselling, relaxation, problems solving, assertiveness training, stress awareness, self report diary, physical exercise, cognitive behavioural therapy, education, relationship orientated therapy, computerised CBT, psychodynamic therapy, cognitive analytic therapy, music making, humour, increasing employees participation and control, clarify role and responsibilities, increase job related information, feedback from supervisors about performance, training and feedback to managers, education training for primary care physicians to attune them to previously unrecognised or untreated anxiety, coping skills, inner quality management training consisting of changing interpretive styles to affect mood and stress, enhancing communication and goal clarity, creating a caring culture and job satisfaction, and quantum management: operationalsing the tools daily. Organisation development interventions consisting of 2 years of surveys, interest groups, action planning and policy intervention and review, meditation, online support group, self management, training in emotional intelligence, and telephone support from supervisor	31 Not reported

Michie and Williams 2003 (1987–1999)	Reducing work related psychological ill health and sickness absence	PP	O,I	I,O	Systematic review	Physical activity, skill training to increase social support and problem solving, early referral to occupational health, and communication training	6 3 on psychological distress, 2 on mental health, 4 on depression, 3 on anxiety, 2 on absenteeism

Mimura and Griffiths 2003 (1990 onwards)	The effectiveness of current approaches to workplace stress management in the nursing profession	PP ?	O,I	I	Systematic	Personnel support, and environmental management interventions	11 Not reported

Edwards and Burnard 2003 (1966–2000)	Systematically identify stressors, moderators and stress management interventions for mental health nurses	PP	O,I	O,I	Systematic	Support, education, awareness, training, ward reorganisation, and behaviour training	77 Not reported

Van der Klink et al. 2001 (1966–1996)	Effectiveness of individual interventions such as CBT, relaxation, multimodal programmes, and organisation focused interventions	PP	O,I, O+I	I,O I+O	Meta analysis	CBT, relaxation, and multimodal and organisation-focused interventions to increase control or support	48 36 on anxiety symptoms, 13 on depressive symptoms, and 7 on absenteeism

Parkes and Sparkes, 1998 (not reported)	(1) effectiveness of individual targeted (2) organisational targeted interventions	PP	O,I	I,O	HSE contract research report. Literature review partially systematic	Individual stress management to change individuals ability to cope with stress and organisation focused interventions to reduce the stress exposure in work environment	9 org studies Not reported

Van der Hek and Plomp, 1997 (1987–1994)	Evaluate the effectiveness by level of intervention and outcome measure	PP	I,O, I+O	I,O,	Systematic review	Individual interventions: relaxation, meditation, biofeedback, cognitive coping strategies, employee assistance programmes Individual-organisational interface: relationships at work, person-environment fit, role issues, worker participation, and autonomy Organisation: Organisation structure, selection and placement, training physical environment, job resign, health concerns, and resources	24 4 on stress, 5 on anxiety, 6 on depression, 3 on absenteeism, and 1 on stress

Murphy, 1996 (1974–1994)	Stress management in work settings: A critical review of the health effects	PP	I	I,O	Systematic	Progressive muscle relaxation, mediation, biofeedback, cognitive behavioural, multimodal, and miscellaneous	64 18 on anxiety, 7 on psychiatric symptoms, 20 on stress, 6 on depression, 7 on absenteeism, and 1 on psychological distress

Saunders et al., 1996 (1977–1991)	The effect of individual stress inoculation training for anxiety and performance	PP	I	I,O	Meta analysis	Stress inoculation training: conceptualisation and education, skill acquisition and rehearsal then application and follow through	37 7 on psychological distress, 9 on test anxiety, 2 on speech anxiety, 1 on computer anxiety, 1 on math anxiety, 6 on teacher stress, 1 on vocational stress, 1 on induced stress, and 1 on social anxiety

Giga et al., 2003 (1990–2001)	The impact of stress management interventions on the individual and the organisation based on UK-based research only	PP	I,O	I,O	Literature review	Individual: relaxation, CBT, exercise, time management, and employee assistance programme Individual/organisational: coworker support groups, role issues, participation and autonomy Organisational: physical and environmental, characteristics, communication, and job redesign/restructuring	16 5 on depression, 7 on absenteeism, 4 on stress, 4 on anxiety

Caulfield et al., 2004 (1993–2003)	To investigate empirical research into occupational stress interventions conducted in Australia	PP	I,O	I,O	Systematic review	Individual: self-management training, stress management Organisational: critical incident stress debriefing (CISD), education programme, job redesign	1 on psychological health, 1 on stress, and 1 on psychological distress

Penalba, McGuire, Leite, 2009 (electronic searches on 12/5/08 Hand searches 1973–1990)	Psychosocial interventions for preventing psychological problems in law enforcement officers (police and military policy), regardless of age, gender, and country	PP SP	I	I	Cochrane review: systematic review of trials	Exercise, psychological interventions, and psychosocial interventions	19 studies reviewed, and only five contained data 3 were exercise based interventions and 7 to psychological interventions Only two studies had psychological measures as outcome

Martin 2009 Published 1997–2000	Meta-analysis of effects of health promotion intervention in the workplace on depression and anxiety symptoms	PP+SP	I+O	I	Meta-analysis	Aerobic and weight training exercise behaviour modification Stress management: extensive and brief CBT, computerised Acceptance and commitment therapy for coping Promotion programme Problem solving, motivational interviewing, ergonomic and job stress, health promotion during physician, consultations, mailed advice, meditation, supervisor committee, feedback, empowerment, quitting strategies, and alcohol reduction	22 studies 17 included in meta-analysis for 20 intervetnion/control comparisons

Conn 2009 (electronic searches 1969–2007)	Meta-analysis of physical activity interventions	PP	I+O	I+O	Meta-analysis of workplace physical activity studies	Intervention including workplace employee, whether worksite designed intervention, during employee's paid time, fitness facilities at worksites, organisational policy present or not, motivational or educational sessions included? Pre post, post post, control intervention comparisons were made. Including not for profit and for profit organisations. Education, health services, government, and manufacturing were the main company types	51 studies delivered interventions at the workplace, and 17 did not 6 studies reported on organisational policy change 38 papers on fitness facilities at worksite 27% supervised exercise and 80% used motivational or educational session

Van Wyk (2010) Searches to 2008, from earliest date of each database	Preventive staff support interventions for health workers	PP	I+O	I+O	Cochrane review	Support groups for staff Training in stress management techniques Management interventions for supporting staff	Ten studies included 2 studies assessed effects of management interventions on absenteeism Yamagishi (2008) reports on career identity intervention on anxiety Von Bayer (1983) 3 sessions of stress management training on state and trait anxiety

Noordik (2010) (searches range from 1966–2007)	Exposure in vivo containing interventions to improve work functioning of worker with anxiety disorders	SP	I	I+O	Systematic review	Comparison of work-based in vivo exposure versus medication, relaxation and response prevention, CBT without exposure, waiting list treatment, imaginal or interceptive exposure, and placebo care as usual (difficult to know if absenteeism affected as reported with other measures of work role)	7 articles included 4 studies on OCD and 1 study on OCD and phobia 2 OCD interventions included in a meta-analysis (Foa, 1984; Aigner, 2004) Studies included in this review as OCD is an anxiety disorder

Cancelliere et al., 2011 (1990–2010)	To determine if workplace health promotion (WHP) programmes are effective in improving presenteeism	PP	I,O	O	Systematic review	Organisational: worksite exercise, a supervisor education program on mental health promotion, “A Lifestyle Intervention Via Email” (Alive!), extra rest break time for workers engaged in highly repetitive work, a multidisciplinary occupational health programme, a multicomponent health promotion programme, participatory processes, exposure to blue-enriched light (versus white light), and a telephone intervention program for depressed workers	14 studies on presenteism

RCTs = randomised controlled trials.

**Table 4:** Effectiveness of SMIs by level and outcome of intervention (results based only on meta-analyses).

Intervention	Individual		Organisational		Mixed/unspecified	
Outcome	Individual	Organisational	Individual	Organisational	Individual	Organisational
Parks and Steelman, 2008				Outcome: absenteeism Wellness programme effect size (mean difference between those with and without a wellness programme, weighted for sample size) *d* = −0.3, 95% CI: −0.48, −0.22***		

Richardson and Rothstein, 2008 GHQ, STAI, SCL-90, various anxiety measures; TQ4, anxiety measure by Bending 1956	CBT for overall psychological outcomes combined *d* = 1.154** Anxiety *d* = 2.390*** For psychological outcomes combined* d* = 0.507*** Anxiety *d* = 0.611***	Absenteeism *d* = 0.213	Organisational (support groups and participatory action groups) for psychological outcomes combined *d* = 0.134 Mental Health *d* = 0.167	Organisational support groups and participatory action groups for absenteeism *d* = −0.159	Stress *d* = 7.27*** Anxiety *d *= 0.678*** Mental Health *d* = 0.441***	

Bond et al., 2006				More control lead to reduced absenteeism 4 studies* d* = −.11 95% CI: −.15 to −.08 Small and significant More support leads to less absenteeism 1 study* d* = −16 95% CI: −.24 to −.09 Small and significant Communication leads to less absenteeism *d* = −.23 Small-to-medium effect and significant		

Marine et al., 2006 Measures of state and trait anxiety STAI, GHQ, Beck	Person-directed interventions versus control: State anxiety: WMD = −9.42, CI: −16.92 to −1.93 Trait anxiety WMD = −6.91; 95% CI: −12.80, −1.01 Findings sustained in medium term: State anxiety: WMD = −0.831, CI: −11.49 to −5.13 Trait anxiety: WMD = −4.09, CI: −7.6 to −0.58 GHQ: person directed interventions did not reduce symptoms: WMD = −11.87, CI: −27.24 to 3.49		GHQ symptoms reduced following combination of knowledge skills training, programme planning WMD: −2.9, CI: −5.16 to −0.64. Anxiety not measured Other single studies using SCL and GHQ did not change results			

Van der Klink et al., 2001 Not listed	CBT on anxiety *d* = 0.70*** Relaxation on anxiety *d* = 0.25* Summation on anxiety *d* = 0.54*** CBT on depression *d* = 0.23 Relaxation on depression *d* = 0.11 Summation on depression *d* = 0.33**	CBT on absenteeism *d* = −0.18 Relaxation on absenteeism *d* = −0.09 Summation on absenteeism *d* = −0.12	Depression *d* = 0	Absenteeism* d* = 0	Anxiety *d *= 0.50*** Depression *d* = 0.59***	

Saunders et al., 1996 Measures of state or trait anxiety, STAI. Others not listed	Performance anxiety *r* = 0.509** State anxiety *r* = 0.373**					

Penalba, McGuire, Leite, 2009 (electronic searches on 12/5/08 Hand searches 1973–1990)	One primary prevention study Backman (1997): mental imaging training versus control					

SCL-90 depression subscale GHQ	Depression outcome: MD (fixed effect) −2.14, CI: −4 to −0.28 at end point (in favour of intervention) 18 months later: MD = −0.97, CI: −2.43 to 0.49 GHQ outcome: MD = 2.74, CI: 0.78–4.7 (in favour of control) 18 month followup: MD = 1.3, CI: −0.61 to 3.21 One secondary prevention study intervention versus control: Norvell (1993) Depression: MD= −7.32, CI: −11.79 to −2.85 in favour of intervention Anxiety: MD = −3.1, CI: −6.94 to 0.6 in favour of intervention All prevention versus control post hoc selected outcomes: Depression: (Backman, 1977; Norvell, 1993) Depression outcome: MD = −0.8, CI: −1.36 to −0.24, in favour of intervention					

Martin 2009 Published 1997–2000 Standardised measures, anxiety, depression and composite measure CES-D BSI	Depression: SMD = Small but significant effect: Depression: SMD = 0.28, CI: 0.12–0.44 Anxiety: SMD = 0.29, CI: 0.06–0.51		Single trial of stress management programme: depression: SMD = 0.69		Depression: SMD:0.31, CI: 0.1–0.51 Anxiety SMD: 0.25, CI: 0.03–0.53 Improvement maintained at followup (only 9 of 17 studies report followup) 5 studies had different methods: Depression in smoking cessation trial: worsening SMD = −0.08 (on CESDscale), no difference in BSI outcomes of depression: SMD = 0.03, anxiety SMD = 0.05	

Conn 2009 (electronic searches 1969–2007) Mood (self report-measure not reported) Work attendance					Unclear which studies that were reported contributed to the effect sizes, and whether they used individual or organisational interventions Mood 2 group post test: mean of effect size (MES): 0.13, CI: −0.05 to 0.31 (NS) 2 group pre- and posttest:MES = 0.21, CI: 0.07 to 0.36** Treatment pre and post: MES + 0.31, CI: 0.22 to 0.4***	Work attendance: 2 group post test: MES = 0.19, CI: 0.11 to 0.27*** 2 group pre post test: MES = 0.05, CI: −0.19 to 0.29 Treatment pre post test: MES 0.02, CI: −0.08 to 0.13 (NS) Workplace interventions had better results, as did intervention in paid time, studies with onsite fitness facilities

Van Wyk (2010) State and trait anxiety index			Career identity training in one study does not improve anxiety in nurses: mean difference: −0.06, CI: −0.44 to 0.32 Von Baeyer's 3 session stress management training showed marginal benefit. Standardised mean difference: −1.45, CI: −2.67 to 0.22	Weir (1997) assessed effect of management intervention to improve process consultation between nurse managers and staff on mean hours absence of staff in a community hospital No difference: mean difference = 20.35, CI: −10.65 to 51.35		

Noordik (2010) Specific outcomes not listed Absenteeism not given as separate outcome from work function	Effects on Anxiety 2 studies in meta-analysis, SMD = −0.54, CI: −1.26 to 0.16 Group exposure CBT and medication versus only medication: SMD = 0.87, CI: 0.34 to 1.39 Exposure in vivo and medication versus only medication: 1, CI: 0.52 to 1.49 Large effect sized (>0.8) judged to indicate significant result without formal statistical tests					

KEY: *d* = effect size, SMD = standardised mean difference, WMD = weighted mean difference, CI = confidence interval. ***P = 0.001, **P = 0.01, *P = 0.05.

When intervention types are not specified the intervention summed in the respective cell are multiple and too many to list. Bold denotes a statistically significant outcome.

**Table 5:** Studies of interventions reaching narrative conclusions without meta-analyses of effect sizes.

Outcome	Number of studies, date range, and key objective of review	MH ↑	MH↓	MH*↔*	A↑	A↓	A*↔*	Narrative conclusions
Egan et al., 2007	18 studies 1981–2006 Psychosocial and health effects of workplace reorganisation Organisational level interventions to improve employee control	6	1	8	4		1	Some evidence of improved mental health as employee control increases and, less consistently, when demands decrease

Lamontagne et al., 2007	90 studies 1990–2005 Job stress intervention literature	**20**	1	20	**21**	**1**	8	Individual focussed, low rates systems approaches are effective at the individual level on anxiety and depression Organisationally focussed high and moderately rated systems approach interventions for job stress show favourable impacts at both organisational and individual levels. Of high rated studies, almost all showed decline in absenteeism

The BOHRF, 2005	19 experimental studies 12 nonexperimental studies Up until April 2004 Workplace interventions for people with common mental health problems	**17**	**1**	4	3		1	Early psychological interventions, including CBT and a range of stress management interventions, are effective for common mental health problems Individual stress management approaches were effective and preferable to multimodal interventions for reducing stress CBT effective for sickness absences associated with common mental health problems

Edwards and Burnard, 2003	70 studies 1966–2000 Effectiveness of stress management for nursing in UK	**5**	1		1			Six stress management intervention studies in UK and one in The Netherlands show that training in behavioural techniques improved levels of sickness in psychiatric nurses. Levels of psychological distress reduced following a 15 week training course in therapeutic skills A great deal is known about stress at work, how to measure it, and impact on many outcomes. However, we lack research into the impact of interventions that attempt to moderate, minimise, or eliminate stressors

Murphy, 1996	64 studies 1974–1994 Health impacts of worksite stress management interventions	**31**		**4**	**5**	**1**		CBT was more effective for psychological outcomes, Combination of techniques, muscle relaxation plus CBT seemed more effective across different outcomes

Giga, 2003	16 studies 1990–2001 Types of stress management interventions used reduce stress in UK over a decade	**10**			**1**		1	Programs that target the individual level were less likely to have an impact on organisational measures Organisational and individual-organisational interventions lead to improvements in health and organisational performance Individual programs were associated with improvements in mental and emotional well-being Action researchers felt the level of intervention was not important but that combination of strategies that addressed employees' needs was critical

Van der Hek and Plomp, 1997	24 studies 1987–1994 Occupational stress management programmes	**8**			**1**		2	It is impossible to recommend which techniques or interventions are most effective and should be recommended There is some evidence that organisationa1 interventions show the best results at the individual-organisational interface and on organisational performance

Mimura and Griffiths, 2003	10 studies 1990 onwards Work place stress interventions for nurses	**7**		3				More evidence for personal support rather than environmental management for workplace stress Cannot answer which approach is more effective

Parkes and Sparkes, 1998	9 studies (dates not reported) All case studies of participatory action research			3			6	Studies difficult to interpret showing ambiguous findings for impact of individual or organisational interventions

Michie and Williams, 2003	6 intervention studies 1987–1999 Reducing work related psychological illness and sickness absence	**2**		1	2			Interventions that improve psychological health and reduce sickness absence used training and organisational approaches to increase participation in decision making and problem solving, increasing support, feedback, and improved communication

Caulfield et al., 2004	6 interventions studies 1993–2003 All peer-reviewed empirical research on occupational stress interventions conducted in Australia in 10 years	**2**		1				Interventions have been primarily individually rather than organisationally focused. Only one was organisationally focused Overall, individually focused interventions do not seem to perform particularly well at lowering work stress Critical incident stress debriefing (CISD) has produced mixed results. On the other hand, seminar-based programmes appear to produce better outcomes

Cancelliere et al., 2011	14 studies 1990–2010 Workplace health promotion (WHP) in improving presenteeism				10	4		Exercise is beneficial in improving presenteeism (not known which specific type of exercise) WHP interventions should be long, intense and frequent and should be based on theory (e.g., behaviour change model) WHP should include physical activity, ergonomic changes and multi-dimensional prevention programmes

MH = mental health, including measures of depression, anxiety, stress, psychosomatic disorder and symptoms, psychiatric symptoms, GHQ; excluding emotional well being or not, and measures of capabilities.

A = absenteeism.

↑ evidence of improvement, ↓ evidence of deterioration, *↔* no evidence of change.
